# Phomaketide A Inhibits Lymphangiogenesis in Human Lymphatic Endothelial Cells

**DOI:** 10.3390/md17040215

**Published:** 2019-04-06

**Authors:** Huai-Ching Tai, Tzong-Huei Lee, Chih-Hsin Tang, Lei-Po Chen, Wei-Cheng Chen, Ming-Shian Lee, Pei-Chi Chen, Chih-Yang Lin, Chih-Wen Chi, Yu-Jen Chen, Cheng-Ta Lai, Shiou-Sheng Chen, Kuang-Wen Liao, Chien-Hsing Lee, Shih-Wei Wang

**Affiliations:** 1 School of Medicine, Fu-Jen Catholic University, New Taipei City 242, Taiwan; taihuai48@hotmail.com; 2 Department of Urology, Fu-Jen Catholic University Hospital, New Taipei City 242, Taiwan; 3 Institute of Fisheries Science, National Taiwan University, Taipei 106, Taiwan; thlee1@ntu.edu.tw (T.-H.L.); d301101010@tmu.edu.tw (M.-S.L.); 4 Chinese Medicine Research Center, China Medical University, Taichung 404, Taiwan; chtang@mail.cmu.edu.tw; 5 Department of Pharmacology, School of Medicine, China Medical University, Taichung 404, Taiwan; 6 Department of Biotechnology, College of Health Science, Asia University, Taichung 413, Taiwan; 7 Department of Orthopaedics, MacKay Memorial Hospital, Taipei 104, Taiwan; robert82ccc@gmail.com (L.-P.C.); wchena7648@gmail.com (W.-C.C.); 8 Ph.D. Degree Program of Biomedical Science and Engineering, National Chiao Tung University, Hsinchu 300, Taiwan; liaonms@pchome.com.tw; 9 Department of Medicine, Mackay Medical College, New Taipei City 252, Taiwan; 1622689043new@gmail.com (P.-C.C.); p123400@hotmail.com (C.-Y.L.); 10 Department of Medical Research, MacKay Memorial Hospital, New Taipei City 251, Taiwan; d48906003@yahoo.com.tw (C.-W.C.); chenmdphd@gmail.com (Y.-J.C.); 11 Department of Radiation Oncology, MacKay Memorial Hospital, Taipei 104, Taiwan; 12 Division of Colon and Rectal Surgery, Department of Surgery, MacKay Memorial Hospital, Taipei 104, Taiwan; laichengta@gmail.com; 13 Division of Urology, Taipei City Hospital HepingFuyou Branch, Taipei 100, Taiwan; DAB67@tpech.gov.tw; 14 Commission for General Education, National United University, Miaoli 360, Taiwan; 15 Institute of Molecular Medicine and Bioengineering, National Chiao Tung University, Hsinchu 300, Taiwan; 16 Department of Biotechnology and Bioindustry Sciences, National Cheng Kung University, Tainan 300, Taiwan; 17 Department of Pharmacology, Graduate Institute of Medicine, College of Medicine, Kaohsiung Medical University, Kaohsiung 807, Taiwan; 18 Department of Medical Research, Kaohsiung Medical University Hospital, Kaohsiung 807, Taiwan; 19 Graduate Institute of Natural Products, College of Pharmacy, Kaohsiung Medical University, Kaohsiung 807, Taiwan

**Keywords:** phomaketide A, lymphangiogenesis, lymphatic endothelial cells, vascular endothelial growth factor receptor-3

## Abstract

Lymphangiogenesis is an important biological process associated with cancer metastasis. The development of new drugs that block lymphangiogenesis represents a promising therapeutic strategy. Marine fungus-derived compound phomaketide A, isolated from the fermented broth of *Phoma* sp. NTOU4195, has been reported to exhibit anti-angiogenic and anti-inflammatory effects. However, its anti-lymphangiogenic activity has not been clarified to date. In this study, we showed that phomaketide A inhibited cell growth, migration, and tube formation of lymphatic endothelial cells (LECs) without an evidence of cytotoxicity. Mechanistic investigations revealed that phomaketide A reduced LECs-induced lymphangiogenesis via vascular endothelial growth factor receptor-3 (VEGFR-3), protein kinase Cδ (PKCδ), and endothelial nitric oxide synthase (eNOS) signalings. Furthermore, human proteome array analysis indicated that phomaketide A significantly enhanced the protein levels of various protease inhibitors, including cystatin A, serpin B6, tissue factor pathway inhibitor (TFPI), and tissue inhibitor matrix metalloproteinase 1 (TIMP-1). Importantly, phomaketide A impeded tumor growth and lymphangiogenesis by decreasing the expression of LYVE-1, a specific marker for lymphatic vessels, in tumor xenograft animal model. These results suggest that phomaketide A may impair lymphangiogenesis by suppressing VEGFR-3, PKCδ, and eNOS signaling cascades, while simultaneously activating protease inhibitors in human LECs. We document for the first time that phomaketide A inhibits lymphangiogenesis both *in vitro* and *in vivo*, which suggests that this natural product could potentially treat cancer metastasis.

## 1. Introduction

Cancer metastasis enables cancer cells to spread from the primary tumor and establish themselves in other tissues. Lymphatic circulation is a common route for cancer metastasis [1], with the thin walls and few tight junctions in the lymphatic vessels offering good permeability. Moreover, the lack of basal lamina and associated pericytes, means that lymphatic capillaries have an easy opening for the uptake of macromolecular cancer cells into the lymphatic vessels [2,3]. Lymphangiogenesis, the process by which new lymphatic vessels grow out of pre-existing vessels, enables lymphatic endothelial cells (LECs) to proliferate and migrate through lymphatic vessels surrounding the tumors [4,5]. Lymphangiogenic factors, including vascular endothelial growth factor (VEGF)-C or D, predominantly bind to VEGF receptor-3 (VEGFR-3), and promote several downstream signaling pathways for regulating lymphangiogenesis [6,7,8]. During the lymphangiogenic process, LEC survival, proliferation, migration and tube formation depends upon the activation of the VEGF-C/VEGFR-3 axis [9]. In various experimental tumor models, overexpression of VEGF-C can induce lymphangiogenesis and disseminate metastatic tumor cells to lymph nodes [10], and the use of neutralizing antibodies against VEGF-C and VEGFR-3 can prevent tumor lymphangiogenesis and lymphatic metastasis [11]. Thus, selective targeting of VEGFR-3-dependent lymphangiogenesis can potentially block cancer progression and metastasis. 

Marine bacteria, microalgae and fungi have proven to be a rich source of bioactive metabolites possessing biological and pharmacological properties with enormous therapeutic potential [12]. We have previously demonstrated that phomaketide A, isolated from the marine endophytic fungal strain *Phoma* sp. NTOU4195, exerts anti-angiogenic and anti-inflammatory effects [13]. However, no data exist as to the effects of phomaketide A on tumor lymphangiogenesis. We therefore explored the *in vitro* and *in vivo* anti-lymphangiogenic effects and mechanisms of phomaketide A.

## 2. Results

### 2.1. Anti-Lymphangiogeneic Effects of Phomaketide A on Human LECs

**Figure 1:**
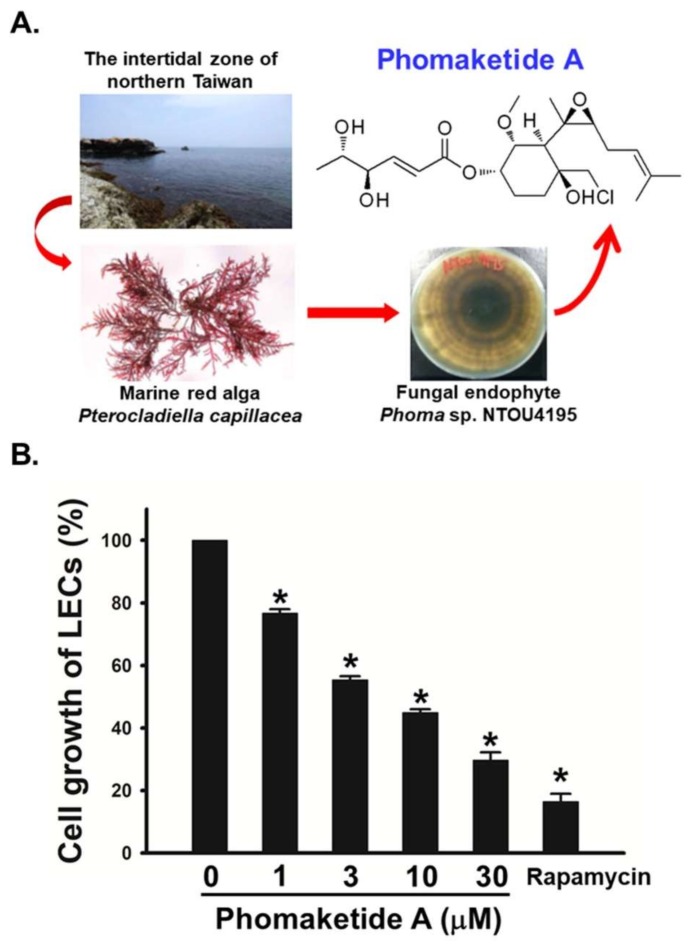
Effects of phomaketide A on cell growth of human lymphatic endothelial cells (LECs). (**A**) Phomaketide A was identified from the fermented broth and mycelium of *Phoma* sp. NTOU4195 isolated from the marine red alga *Pterocladiella capillacea* harvested along the north coast of Taiwan. (**B**) Cells were treated with various concentrations of phomaketide A and rapamycin (10 μM) for 48 h, and anti-lymphangiogenic activity was explored in a cell growth assay (N = 3). Data are expressed as the mean ± SEM. * *p* < 0.05 compared with the control group.

**Figure 2:**
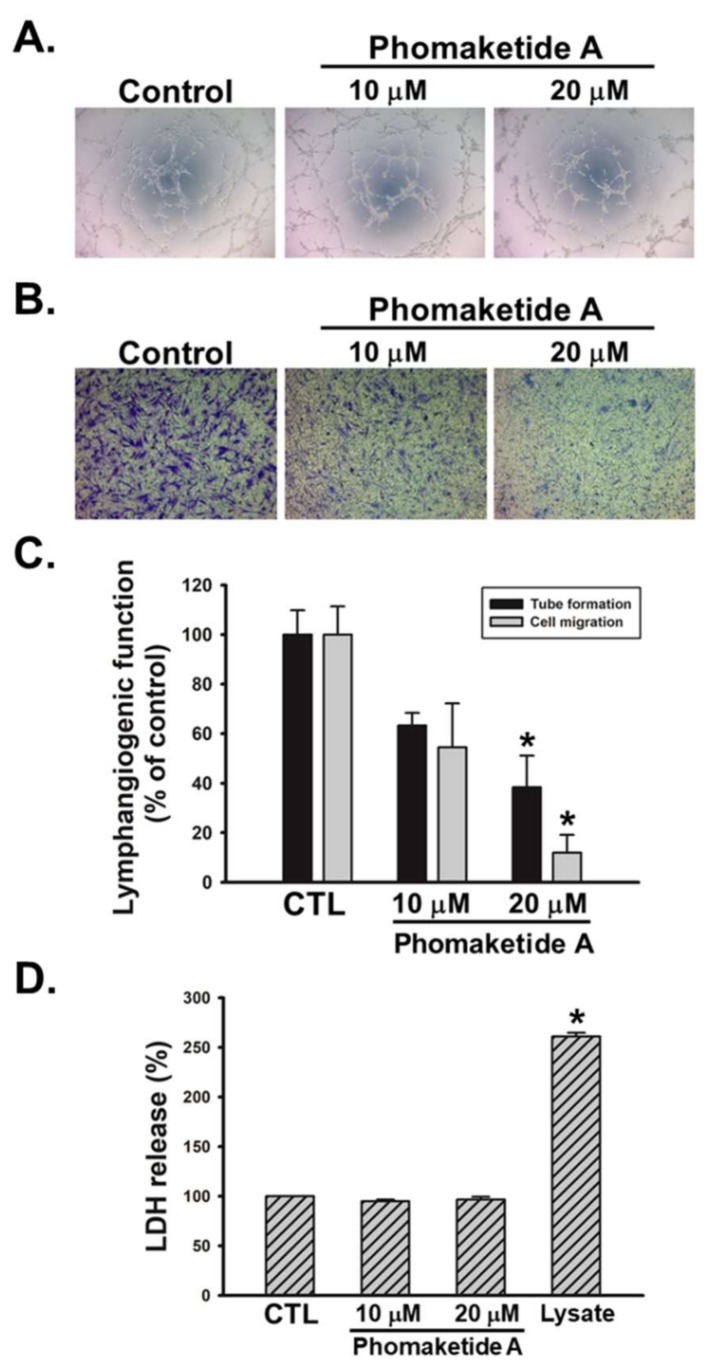
Effects of phomaketide A on human LECs tube formation, migration, and cytotoxicity. Cells were treated with the indicated concentrations of phomaketide A for 8 h. Capillary-like structure formation (**A**) and cell migration (**B**) were examined by tube formation and Transwell assays, respectively (N = 4–6). (**C**) ImageJ software was used to validate the lymphangiogenic functions of phomaketide A. (**D**) Cells were treated with phomaketide A for 24 h, then cytotoxicity was evaluated by lactate dehydrogenase (LDH) assay (N = 3). Data are expressed as the mean ± SEM. * *p* < 0.05 compared with the control (CTL) group.

To examine the growth-inhibitory effects of phomaketide A (Figure 1A), we treated human LECs with various concentrations of phomaketide A. Figure 1B illustrates how phomaketide A inhibited LECs growth in a concentration-dependent manner (IC_50_ = 3.7 ± 0.6 μM). The lymphangiogenesis inhibitor, rapamycin, served as a positive control [14]. Next, the results of a tube formation assay showed that phomaketide A significantly reduced LECs tube formation (Figure 2A). Since the migratory ability of endothelial cells is another essential characteristic of lymphangiogenesis [15], we therefore evaluated the effect of phomaketide A on LECs migration. We found that phomaketide A markedly reduced the numbers of LECs that migrated through the Transwell inserts (Figure 2B). Moreover, there were no discernable increase on the levels of lactate dehydrogenase (LDH) with either dose of phomaketide A as compared with untreated human LECs (controls) (Figure 2D). Thus, phomaketide A appears to exert anti-lymphangiogenic effects without any evidence of cytotoxicity in human LECs.

### 2.2. Phomaketide A Inhibits the VEGFR-3 and PKCδ Signaling Pathway in Human LECs

**Figure 3:**
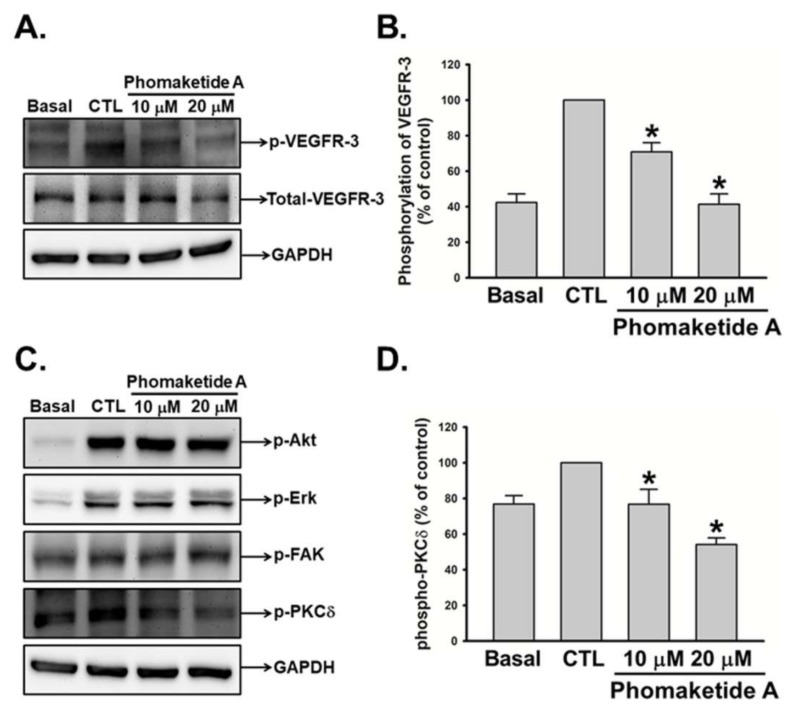
Modulation of phomaketide A on VEGFR-3 and downstream signaling pathways in human LECs. (**A** and **C**) Quiescent LECs were treated with or without EGM-2MV medium in the absence (CTL) or presence of phomaketide A for 5–10 min. The phosphorylation of VEGFR-3, Akt, Erk, FAK, and PKCδ were determined by Western blot analysis (N = 5–7). The quantitative densitometry of the relative levels of phosphorylated VEGFR-3 and PKCδ were measured by Image-Pro Plus processing software (**B** and **D**). Data are expressed as the mean ± SEM. * *p* < 0.05 compared with the control (CTL) group.

To elucidate the mechanisms employed by phomaketide A to regulate lymphangiogenesis, we explored its effects upon VEGFR-3 in LECs. Figure 3A,B illustrate the significant suppression by phomaketide A upon the phosphorylation of VEGFR-3. We also investigated the effects of phomaketide A upon the signal transduction downstream of VEGFR-3. The results indicated that phomaketide A dramatically decreased the phosphorylation of PKCδ, but did not affect the phosphorylated levels of Akt, Erk, or FAK in the LECs (Figure 3C,D). Our results demonstrate that phomaketide A reduces lymphangiogenesis through VEGFR-3 and PKCδ-dependent pathways in human LECs.

### 2.3. Phomaketide A Impedes eNOS Activation in Human LECs

**Figure 4:**
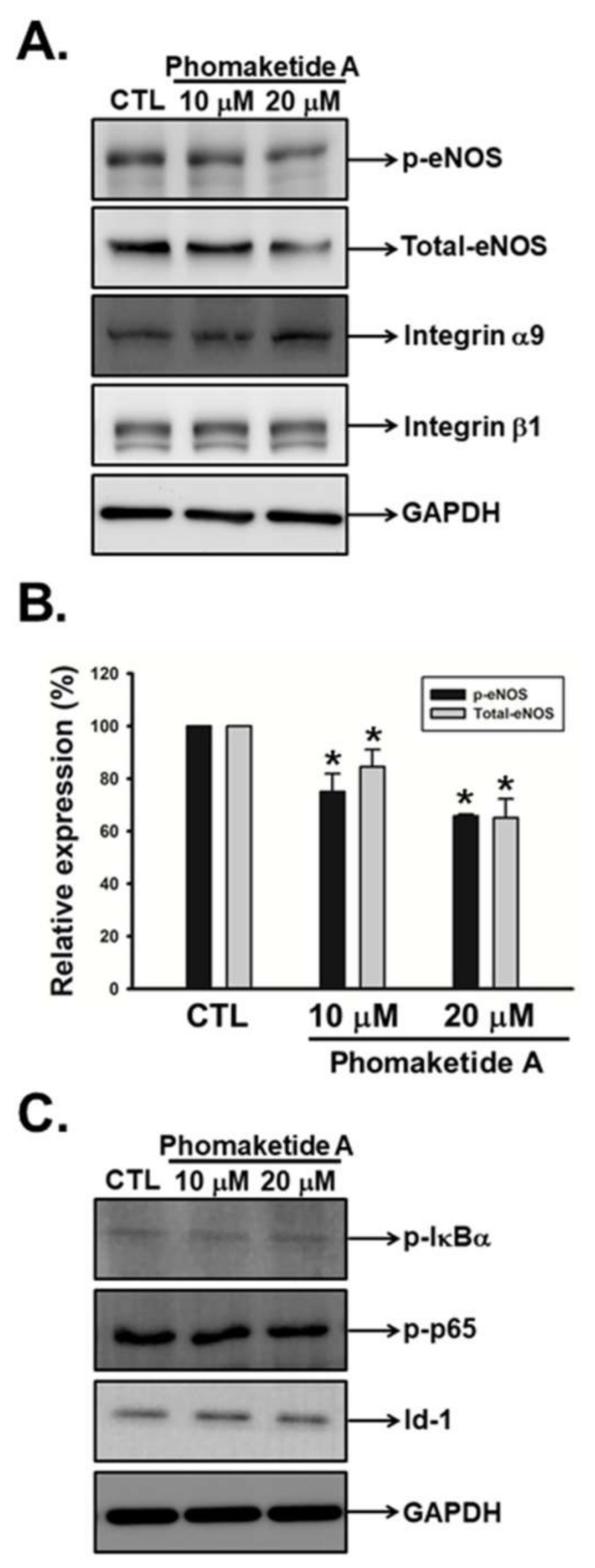
Effects of phomaketide A on pro-lymphangiogenic signals and transcription factors in human LECs. (**A** and **C**) Cells were treated with the indicated concentrations of phomaketide A for 8 h, and the indicated phosphorylated and total proteins were determined by Western blot analysis (N = 4–6). Image-Pro Plus processing software quantified the relative level of protein (**B**). Data are expressed as the mean ± SEM. * *p* < 0.05 compared with the control (CTL) group.

Recent studies have documented that integrin α9, integrin β1, and endothelial nitric oxide synthase (eNOS) were associated with VEGFR-3-mediated lymphangiogenesis [8,16,17]. We therefore investigated whether phomaketide A inhibits LECs-induced lymphangiogenesis via these molecular signals. We found that phomaketide A significantly suppressed the phosphorylation and expression of eNOS but did not impair the protein levels of integrin α9 and β1 (Figure 4A,B). Activation of nuclear factor-κB (NF-κB) and overexpression of Id-1 (inhibitor of differentiation/DNA binding) are critical for multiple physiological and pathological processes, including lymphangiogenesis [17,18,19]. Here, we found that phomaketide A did not alter the phosphorylation of p65 or IκBα, nor the expression of Id-1 in LECs. We suggest that the eNOS signaling pathway is involved in phomaketide A-induced anti-lymphangiogenic effects in human LECs.

### 2.4. Phomaketide A Increases the Expression Profile of Protease Inhibitors in Human LECs

**Figure 5:**
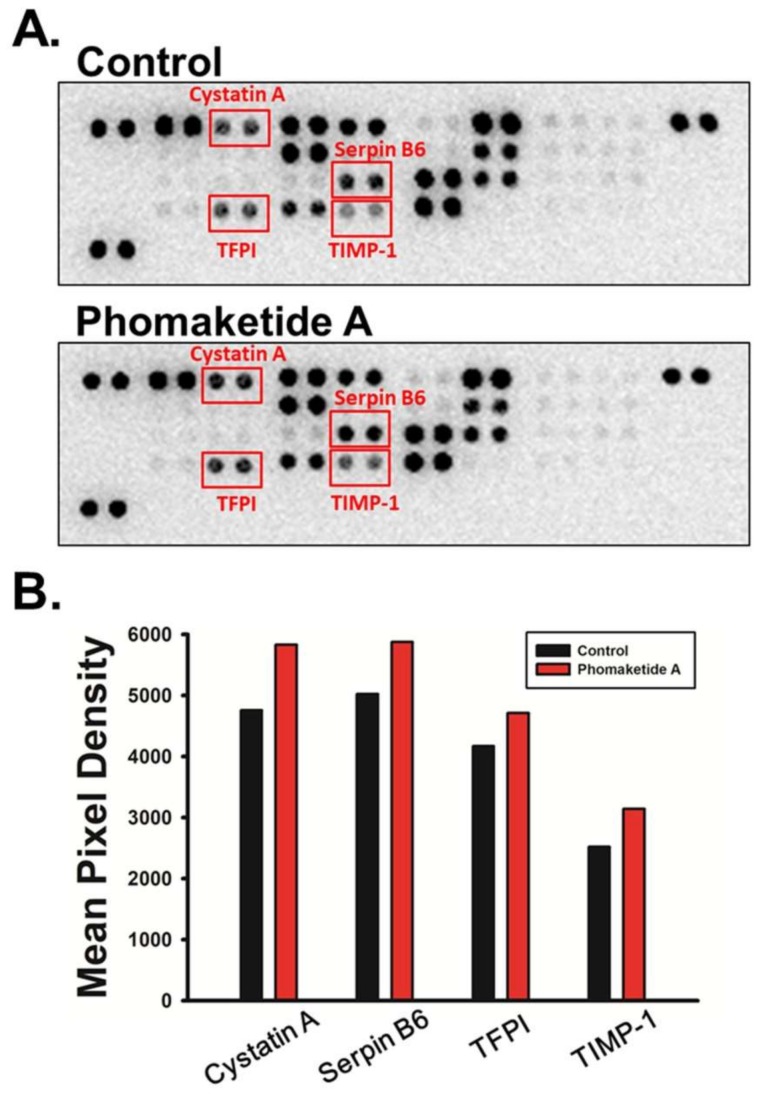
Effects of phomaketide A on protease inhibitor expression profiles in human LECs. (**A**) LECs were treated with phomaketide A (20 μM) for 8 h, then total cell lysates were collected. Significant changes in protein spots detected by human protease inhibitor array are indicated. (**B**) Profiles of mean spot pixel densities for upregulated protease inhibitors were analyzed using ImageJ software.

Proteolytic activities of cells are coordinately regulated by proteases and protease inhibitors in a coordinated fashion for the degradation of the extracellular matrix (ECM). Analysis of protease and/or protease inhibitor expression profiles is essential to determine how they affect normal cellular function and dysregulate LECs lymphangiogenesis [20]. Using human proteome arrays, we found that phomaketide A did not affect the relative expression of 35 human proteases (Supplementary Figure 1). However, phomaketide A substantially increased the levels of protein expression of several human protease inhibitors in LECs (Figure 5A); the four most significantly upregulated protease inhibitors were cystatin A, serpin B6, tissue factor pathway inhibitor (TFPI), and tissue inhibitor matrix metalloproteinase 1 (TIMP-1) (Figure 5B). These results indicate that phomaketide A may inhibit lymphangiogenesis via the upregulation of cystatin A, serpin B6, TFPI, and TIMP-1 in LECs.

### 2.5. Phomaketide A Impairs Tumor Lymphangiogenesis in the A549 Xenograft Model

**Figure 6:**
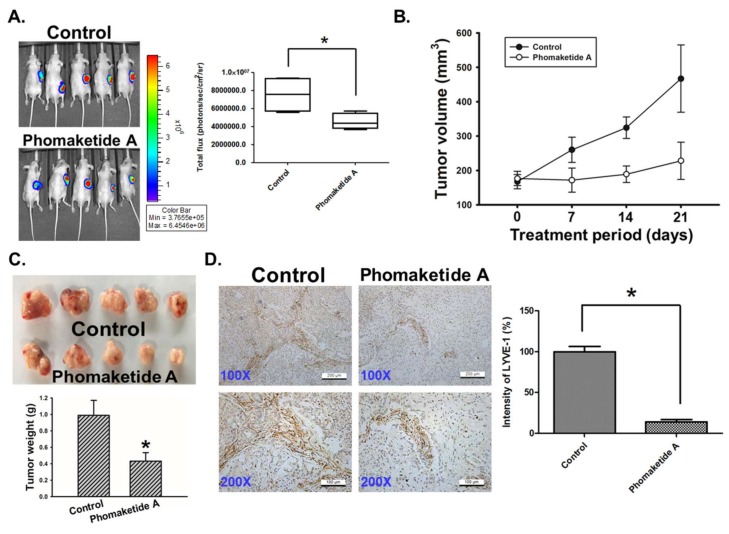
Effects of phomaketide A on tumor-associated lymphangiogenesis. (**A**) A549-Leu cells were injected into flank sites of nude mice for 4 weeks. Then, animals (five mice/group) were given vehicle (control) and phomaketide A (20 mg/kg) by i.p. injection for 3 consecutive weeks. The tumor size was monitored by bioluminescence imaging. Representative IVIS images of tumor growth and quantitative analysis of imaging signal intensity was seen on day 21. (**B**) Treatment period was indicated (days 0–21), and tumor volume was measured manually every week. (**C**) A549 tumor samples from animals were obtained and weighted at the end of the treatment. (**D**) The xenograft tumors were excised and stained with lymphatic vessel marker LYVE-1 by IHC analysis (N = 4). Representative images of LYVE-1 expression in tumor specimens are seen. The quantification of LYVE-1 expression was analyzed using ImageJ software. Data are expressed as the mean ± SEM. *, *p* < 0.05 compared with the control group.

To validate *in vivo* significance of the cellular observations, we determined the effect of phomaketide A on tumor xenograft growth. As shown in Figure 6A, phomaketide A repressed A549 tumor growth via a remarkable reduction of bioluminescence activity. Furthermore, we found that phomaketide A dramatically decreased the tumor volume and weight of A549 xenografts (Figure 6B,C). Immunohistochemical analysis revealed that the expression of LYVE-1, the key specific marker for lymphatic vessels [21], was obviously decreased by phomaketide A (Figure 6D). Based on these findings, we propose that phomaketide A blocks *in vivo* tumor progression by suppressing lymphangiogenesis.

## 3. Discussion

Several studies have shown that marine-derived compounds possess biological activity and pharmacological effect in cancer models with little or no side effects [22,23,24]. To our knowledge, this is the first study illustrating the anti-lymphangiogenesis property of phomaketide A. Marine organisms are the cradle for many excellent pharmaceutical products, particularly in anti-lymphangiogenesis. For example, fucoidan from *Undaria pinnatifida* sporophylls exerts anti-metastasis and anti-lymphangiogenesis activities by reducing HIF-1α/VEGF-C resulting in attenuation of the PI3K/Akt/mTOR signaling pathways [25]. In addition, toluquinol, isolated from the culture broth of the marine fungus *Penicillium* sp. HL-85-ALS5-R004, has been reported to suppress lymphangiogenesis by down-regulating the VEGF-C/VEGFR-3 cascade [26]. A recent study has shown that tuberazines C obtained from Taiwanese zoanthid *Palythoa tuberculosa* displayed the anti-lymphangiogenesis effect through the suppression of cell growth and tube formation in human LECs [14]. Here, we discover that phomaketide A, isolated from the marine endophytic fungal strain *Phoma* sp. NTOU4195 [13], possesses the anti-lymphangiogenic function against human LECs. Marine-derived natural product phomaketide A has the potential to impede tumor-associated lymphangiogenesis and metastasis. 

VEGFR-3 has been reported to play an important role in lymphangiogenesis and tumor invasion through the lymphatics [27]. On stimulation by its ligand VEGF-C, VEGFR-3 was phosphorylated which promotes the proliferation and migration of LECs resulting in lymphangiogenesis [9]. Previous studies showed that Ki23057 inhibits phosphorylation of VEGFR-3 against the lymphangiogenesis of gastric cancer [27]. In the current study, we demonstrated that phomaketide A significantly inhibited the phosphorylation of VEGFR-3 in human LECs. Moreover, PKC δ is associated with the phosphorylation of VEGFR-3 in endothelial cells [28]. Our results found that phomaketide A also induced the reduction of PKCδ phosphorylation. PKCδ mediated by VEGFR-3 phosphorylation underlying phomaketide A treatment deserves further investigation. In addition, eNOS is involved in VEGF-C-induced lymphangiogenesis and plays a vital role in lymphatic metastasis [8]. Doxycycline can decrease eNOS phosphorylation to inhibit LECs proliferation [29]. Pro-inflammatory cytokines, IL-20 and IL-33, also activate the phosphorylation of eNOS to promote the proliferation, migration and tube formation of LECs [30,31]. In the current study, phomaketide A suppressed the phosphorylation and expression of eNOS in LECs, confirming the importance of eNOS in LECs-regulated lymphangiogenic processes.

Proteolytic processing of VEGF-C regulates lymphatic vessel growth for activating LECs lymphangiogenesis [32,33], whereas lymphatic vessel outgrowth is reduced by protease inhibitor [34]. Several studies demonstrate that serine proteinase inhibitor (serpin) functioned as a lymphangiogenesis inhibitor to suppress the lymphatic metastasis of cancer and the LECs proliferation and migration [35,36,37]. The expression of tissue inhibitor of metalloproteinase-2 (TIMP-2) is lower or negative in patients with lymph node metastasis [38]. Furthermore, matrix metalloproteinases inhibitors are well recognized to impair lymphangiogenesis [39]. Our results showed that anti-lymphangiogenic effects of phomaketide A were associated with the induction of protease inhibitors such as cystatin A, serpin B6, TFPI and TIMP-1. Meanwhile, several studies have demonstrated that promotion of these protease inhibitors can induce anti-angiogenic effects [40,41,42,43]. Our previous report reveals that phomaketide A is a novel angiogenesis inhibitor [13]. Phomaketide A is a potential dual-effect agent that could be used for cancer treatment through the inhibition of angiogenesis and lymphangiogenesis.

**Figure 7:**
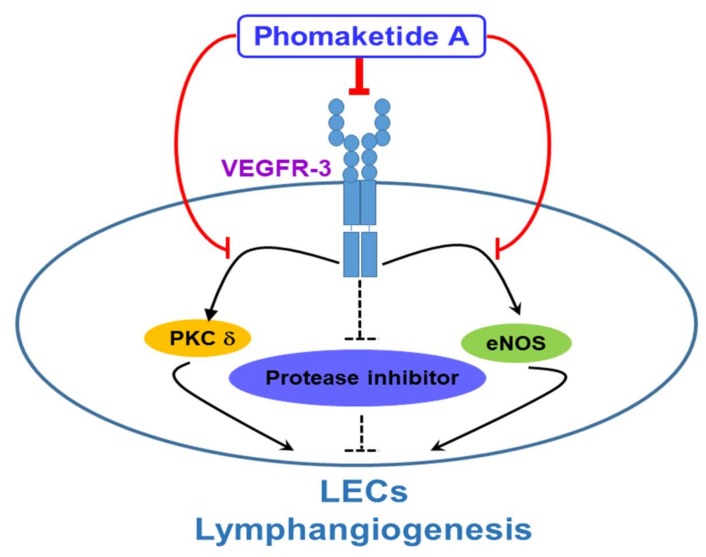
Schema of phomaketide A-induced anti-lymphangiogenic mechanism in human LECs. This study reveals phomaketide A as a promising anti-lymphangiogenic agent. Phomaketide A may inhibit lymphangiogenesis by decreasing VEGFR-3 and its downstream PKCδ and eNOS signaling pathways, as well as increasing protease inhibitors in human LECs.

In conclusion, this report discloses a novel mechanism by which phomaketide A reduces LECs lymphangiogenesis *in vitro* and *in vivo*. We demonstrate that phomaketide A antagonizes lymphangiogenesis by decreasing VEGFR-3 and its downstream PKCδ and eNOS signaling pathways, as well as increasing protease inhibitors in human LECs (Figure 7). Our previous study demonstrates promising *in vitro* effects with phomaketide A in human EPCs. In this study, we show that phomaketide A also exerts anti-lymphangiogenic effect in LECs (IC_50_, 3.7 ± 0.6 µM), and profoundly inhibit the proliferation of A549 lung cancer cells, with an IC_50_ value of 3.0 ± 0.3 μM (Supplementary Figure S2). We therefore suggest that phomaketide A acts selective and potent inhibitory effects upon endothelial cells and A549 cancer cells. Phomaketide A can coordinately induce anti-angiogenic, anti-lymphangiogenic, and anti-cancer effects in A549 tumor microenvironment, resulting in the inhibition of *in vivo* tumor progression. The detailed anti-cancer effects and mechanisms of phomaketide A in A549 cancer cells should be further investigated. LECs have been proposed to dictate tumor lymphangiogenesis and metastasis in microenvironment. Our findings suggest that phomaketide A may serve as a lead candidate for further development for novel lymphangiogenesis inhibitors to block cancer progression and metastasis.

## 4. Materials and Methods

### 4.1. Materials 

Phomaketide A was isolated from the fermentation broth and mycelium of the endophytic fungal strain *Phoma* sp. NTOU4195 as previously described [13]. Rapamycin was purchased from Cayman Chemical (Ann Arbor, MI, USA). The compounds were dissolved in dimethyl sulfoxide (DMSO) at 20 mM as a stock solution for all assays. DMSO was used as the vehicle control in the experiments. DMSO and other chemical agents were obtained from Sigma-Aldrich (St. Louis, MO, USA). Rabbit polyclonal antibody specific for phospho-VEGFR-3 (Tyr1230/1231) was purchased from Cell Application (San Diego, CA, USA). Rabbit monoclonal antibodies specific for phospho-Akt (Ser473), phospho-ERK (Thr202/Tyr204), phospho-FAK (Tyr397), phospho-PKCδ (Thr505), phospho-eNOS (Ser1177), eNOS, Integrin β1, VEGFR-3, and GAPDH were purchased from Cell Signaling Technologies (Boston, MA, USA). Rabbit monoclonal antibodies specific for Integrin α9, phospho-p65 (Ser536) and phospho-IκBα (Ser32) were purchased from Abcam (Cambridge, MA, USA). Rabbit polyclonal antibody specific for Id-1 was purchased from Santa Cruz Biotechnology (Santa Cruz, CA, USA). Gibco-BRL life technologies (Grand Island, NY, USA) supplied Dulbecco’s modified Eagle’s medium (DMEM), fetal bovine serum (FBS), and all other cell culture reagents. Matrigel was obtained from BD Biosciences (Bedford, MA, USA).

### 4.2. Cell Culture

The human lymphatic endothelial cells (LECs) were purchased from Lonza (Walkersville, MD, USA). LECs were cultured in EGM-2MV medium consisting of EBM-2 basal medium and SingleQuots kit (Lonza). A549 cell line was purchased from American Type Culture Collection (Manassas, Virginia, USA). To establish the luciferase-tagged A549 cells (A549-Leu cells), pLenti PGK Blast V5-LUC (w528-1) for expression of the firefly luciferase was purchased from the Addgene (Plasmid #19166; Watertown, MA, USA). Lentiviruses were prepared according to standard protocols. For transduction, 3 × 10^5^ A549 cells were seeded in a 6-well plate and the lentivirus was added (multiplicity of infection = 10) in medium containing polybrene (8 μg/mL). The culture medium was changed after 24 h. The cells were incubated with 5 μg/mL blasticidin for 48 h for stable clone selection. The surviving cells were selected, and clonal cell populations were expanded as A549-Luc cell line. A549-Leu cells were maintained in DMEM, supplemented with 10% fetal bovine serum (FBS). The cells culture conditions are recorded in our previous paper [14].

### 4.3. Cell Growth Assay

LECs were cultured in 96-well plates at a density of 5 × 10^3^ cells in each well. Overnight, the culture medium was replaced with fresh EGM-2MV medium in the presence of either vehicle (DMSO) or phomaketide A for 48 h treatment. The assay on cell growth of LECs was determined by the method based on our previous study [14].

### 4.4. Capillary Tube Formation Assay

The capillary tube formation assay was performed on Matrigel-coated 96-well plates. LECs were seeded at a density of 2 × 10^4^ cells per well and incubated in EGM-2MV medium and the indicated concentration of tested compounds, for 8 h at 37 °C. The detection and quantification of LECs tube formation were examined according to previously described procedures [14].

### 4.5. Cell Migration Assay

The cell migration assay was conducted in Transwell inserts (Corning, NY, USA) with 2.5 × 10^4^ of LECs per well seeded onto the upper chamber with EBM-2 basal medium. The upper chamber was transferred and incubated in the bottom chamber with EGM-2MV medium and the indicated concentration of tested compounds was for 8 h. Cell migration was determined according to our previous protocol [44].

### 4.6. Cytotoxicity Assay

LECs (5 × 10^3^ cells/well) were seeded onto 96-well plates and incubated with EGM-2MV medium in the presence of a vehicle (DMSO) or phomaketide A for 8 h of treatment. Release of lactate dehydrogenase (LDH) into the medium was measured using a cytotoxicity assay kit (Promega, Madison, WI, USA) according to an established protocol [14].

### 4.7. Western Blot Analysis

After the treatment of LECs, the reaction was terminated by the addition of lysis buffer containing a protease inhibitor cocktail (Roche, Mannheim, Germany). Total cell lysates were electrophoresed using SDS-PAGE and subsequently transferred to polyvinylidene difluoride membranes. After blocking the blots with 4% bovine serum albumin, they were treated with primary antibody and then peroxidase-conjugated secondary antibody. The blots were visualized using enhanced chemiluminescence and monitored using the UVP Biospectrum system (UVP, Upland, CA, USA). 

### 4.8. Proteome Profile Arrays

Human protease and protease inhibitor antibody arrays (R&D Systems, Minneapolis, MN, USA) were used to analyze the expression profiles of protease and protease inhibitor according to the manufacturer’s instructions. UVP Biospectrum system was used to detect chemiluminescent signals, which were further analyzed using ImageJ software.

### 4.9. In Vivo Tumor Xenograft Model

All animal procedures were performed according to approved protocols issued by the China Medical University (Taichung, Taiwan) Institutional Animal Care and Use Committees. Male nude mice (4-week of age) were used in the subcutaneous xenograft model. A549-Leu cells (2 × 10^6^ cells) were resuspended in 0.1 mL of 50% serum-free medium and 50% Matrigel, and injected into the right flank of each animal. Four weeks after A549-Leu cells injection, the mice were randomized into experimental and control groups according to bioluminescence imaging from the Xenogen IVIS imaging system 200 (PerkinElmer, MA, USA). Then, the mice were treated with phomaketide A (20 mg/kg) or the vehicle control every other day by intraperitoneal (i.p.) administration (five mice per group). After the treatment, the animals were sacrificed, and the tumor specimens were resected for immunohistochemical (IHC) analysis.

### 4.10. IHC Analysis

The tumor tissues were placed on glass slides, rehydrated and incubated in 3% hydrogen peroxide to block the endogenous peroxidase activity. After trypsinization, nonspecific antibody-binding sites were blocked using 3% bovine serum albumin in PBS. IHC analysis was carried out to determine the expression of lymphangiogenic marker according to the standard protocol [45].

### 4.11. Statistical Analysis

Data points represent the mean ± standard error of mean (SEM). Statistical analyses of data were done with one-way ANOVA followed by Student’s t-test. The difference is significant if the *p* value is < 0.05.

## Supplementary Materials

The following are available online at https://www.mdpi.com/1660-3397/17/4/215/s1, Figure S1: Effect of phomaketide A on the expression profile of proteases in human LECs. Figure S2: Effect of phomaketide A on cell growth of A549 cancer cells.
