# Supplementary material for: Phomaketide A Inhibits Lymphangiogenesis in Human Lymphatic Endothelial Cells
Source: Mar Drugs. 2019 Apr 6;17(4):215. doi: 10.3390/md17040215 (PMC6520718; doi:10.3390/md17040215)
Supplement: Supplementary file 1 [file marinedrugs-17-00215-s001.pdf]

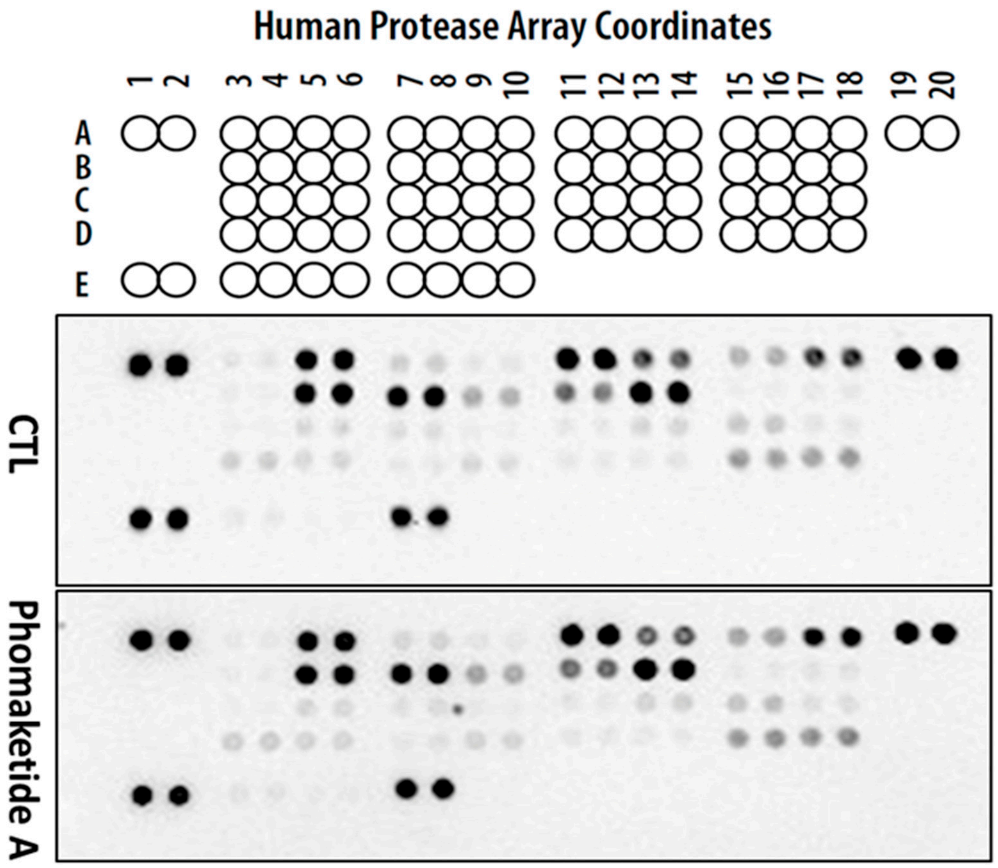

Coordinate	protein	Coordinate	protein	Coordinate	protein	Coordinate	protein
A1, A2	Reference Spots	B3, B4	Cathepsin E	C7, C8	Kallikrein 10	D11, D12	MMP-12
A3, A4	ADAM8	B5, B6	Cathepsin L	C9, C10	Kallikrein 11	D13, D14	MMP-13
A5, A6	ADAM9	B7, B8	Cathepsin S	C11, C12	Kallikrein 13	D15, D16	Neprilysin
A7, A8	ADAMTS1	B9, B10	Cathepsin V	C13, C14	MMP-1	D17, D18	Presenilin
A9, A10	ADAMTS13	B11, B12	Cathepsin X/Z/P	C15, C16	MMP-2	E1, E2	Reference Spots
A11, A12	Cathepsin A	B13, B14	DPPIV	C17, C18	MMP-3	E3, E4	Proprotein Convertase 9
A13, A14	Cathepsin B	B15, B16	Kallikrein 3	D3, D4	MMP-7	E5, E6	Proteinase 3
A15, A16	Cathepsin C	B17, B18	Kallikrein 5	D5, D6	MMP-8	E7, E8	uPA
A17, A18	Cathepsin D	C3, C4	Kallikrein 6	D7, D8	MMP-9	E9, E10	Negative Control
A19, A20	Reference Spots	C5, C6	Kallikrein 7	D9, D10	MMP-10		

Suppl. Figure.1 Effect of phomaketide A on the expression profile of proteases in human LECs. LECs were treated with phomaketide A (20 μM) for 8 h, subsequently total cell lysates were collected to detect the relative levels of protease using human protease array. The relative expression and coordinate of 35 human proteases are shown.

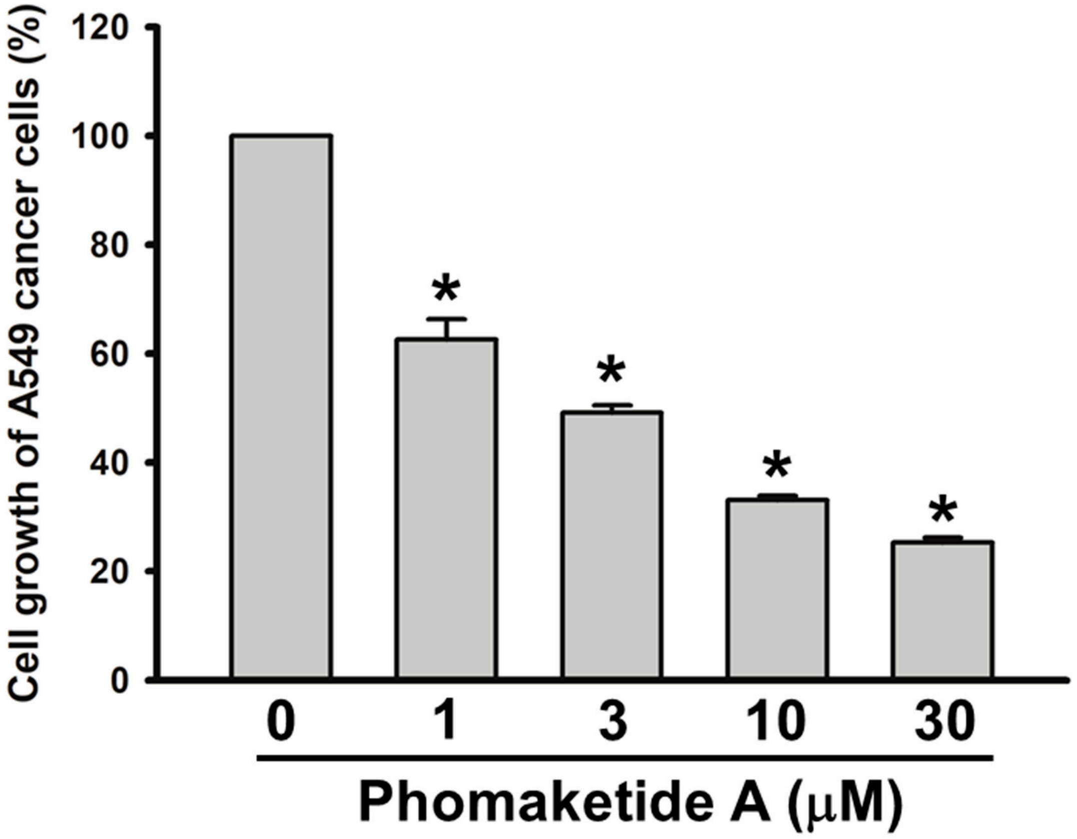

Suppl. Figure.2 Effect of phomaketide A on cell growth of A549 cancer cells. Cells were treated with the indicated concentrations of phomaketide A for 48 h, and anti-cancer activity was determined using cell growth assay. Data are expressed as the mean ± SEM of three independent experiments. * $p < 0.05$, compared with the control group.
